# Automated processing of railway track deflection signals obtained from velocity and acceleration measurements

**DOI:** 10.1177/0954409718762172

**Published:** 2018-03-19

**Authors:** David Milne, Louis L Pen, David Thompson, William Powrie

**Affiliations:** Faculty of Engineering and the Environment, University of Southampton, Southampton, UK

**Keywords:** Accelerometers, geophones, lineside monitoring, performance monitoring, railway track, track displacement, track vibration

## Abstract

Measurements of low-frequency vibration are increasingly being used to assess the condition and performance of railway tracks. Displacements used to characterise the track movement under train loads are commonly obtained from velocity or acceleration signals. Artefacts from signal processing, which lead to a shift in the datum associated with the at-rest position, as well as variability between successive wheels, mean that interpreting measurements is non-trivial. As a result, deflections are often interpreted by inspection rather than following an algorithmic or statistical process. This can limit the amount of data that can be usefully analysed in practice, militating against widespread or long-term use of track vibration measurements for condition or performance monitoring purposes. This paper shows how the cumulative distribution function of the track deflection can be used to identify the at-rest position and to interpret the typical range of track movement from displacement data. This process can be used to correct the shift in the at-rest position in velocity or acceleration data, to determine the proportion of upward and downward movement and to align data from multiple transducers to a common datum for visualising deflection as a function of distance along the track. The technique provides a means of characterising track displacement automatically, which can be used as a measure of system performance. This enables large volumes of track vibration data to be used for condition monitoring.

## Introduction

Motion transducers are increasingly being used to measure the low-frequency vibration of railway tracks to evaluate condition and performance. The main objective is usually to obtain a signal with which to quantify the track movement under a passing train. A range of measurement technologies can be used for this purpose. Track displacement can be measured directly using a high-speed video of the track-mounted targets with digital image correlation,^1,2^ laser-based systems^3–5^ or multi-depth deflectometers installed in the track bed.^6^ Alternatively, geophones or accelerometers may be used to measure the velocity or acceleration. These signals must be filtered and integrated, once for velocity or twice for acceleration, to obtain displacement.^7–10^ Characterising the signal is not straightforward, owing to artefacts from signal processing including the lack of an absolute displacement datum for velocity or acceleration measurements and the variability of the signal arising from differences in the wheel load and dynamic behaviour along a train.

In obtaining a displacement signal from velocity or acceleration measurements, it is necessary to apply a high-pass filter to prevent low-frequency drift.^7^ The content of the displacement signal obtained depends on the filter used. The filter’s cut-off frequency must be high enough to prevent low-frequency drift, so that the signal contains a stationary region, in which there is a regular pattern of deflection from repeating vehicles during a train passage, without filtering out information relevant for the displacement, at and above the vehicle passing frequency.^11,12^ In practice, when using a Butterworth filter, cut-off frequencies between 1/2 and 3/4 (e.g. 2/3) of the vehicle passing frequency have been found to be appropriate, depending on the actual speed of the train and order of the filter.^1,7,13,14^

A low-pass filter is often used to remove higher frequency content not significant for low-frequency track deflection. The contribution of vibration above about 10–12 times the vehicle passing frequency to track deflection is small.^7,11,12,15^ This means that the sample rate needed for obtaining track deflection is about 100 Hz for trains operated in the United Kingdom. However, the sensor output may be oversampled and filtered during acquisition to improve resolution and reduce noise, and data may be stored at higher sample rates to relax filter performance requirements for later signal processing.

The application of the high-pass filter results in low-frequency transients in the displacement signal around the first and last bogies of the train and a shift in the datum associated with the at-rest position (zero deflection) of the track within the stationary region of the signal.^7,16^ The shift in the at-rest datum position means that it is difficult to determine the proportion of the track movement that is upward or downward, or to align results from multiple transducers to a common level. Approaches to alleviate this problem and to improve the quality of vibration data at low frequency, by using different integration schemes and by using notch rather than band-pass filters,^13,17^ have met with limited success.

**Figure 1.**
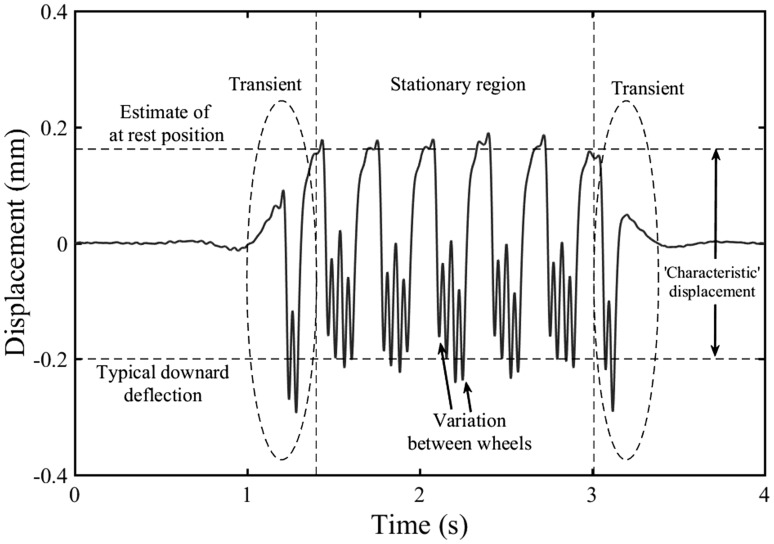
Track displacement obtained from sleeper velocity measurements using geophones, sampled at 500 Hz, indicating artefacts from signal processing, variability between wheels and how the signal might be interpreted by inspection.

Figure 1 shows an example of measurements obtained from a six-vehicle train in which the transients at the start and end of the sequence and the shift in the datum associated with the at-rest position are clearly seen. Also, the deflection under each wheel differs. The pattern and amplitude of the track deflection depends on the sequence of applied wheel loads. There will inevitably be variations between the deflections under each wheel, as a result of differences in the mass and occupancy between vehicles in the train; interaction between adjacent wheels; and the dynamic behaviour of each wheel due to track and wheel unevenness, including impacts from wheel flats. This variability makes it difficult to define explicitly an average or typical deflection; the maximum deflection range would be an overestimate of typical behaviour. Consequently, track displacements are often interpreted by visual inspection.

Generally, the pattern of deflection from a train is relatively uniform as most trains comprise several near-identical vehicles or have a repeating primary vehicle type. This means that an experienced analyst can become quite adept at inferring the location of the at-rest datum and judging the typical range of downward deflection to obtain what is often referred to as the ‘characteristic’ displacement.

Advances in affordable and robust sensor technology mean that railway track vibration measurements could be used for condition monitoring in larger or more long-term applications.^18^ This requires development of automated process for data interpretation, as characterising the signal by inspection would not be feasible for hundreds, let alone thousands of train passages. This paper develops a method for interpreting track displacement data using the cumulative distribution of the deflections. This overcomes artefacts from signal processing, allowing the at-rest position or false datum to be identified and corrected for as required, using a statistical process. The same principle is used to characterise the typical magnitude of track displacement. The method can be implemented to interpret track displacement data automatically using a computer. The approach is also applicable for interpreting direct measurements of track displacement as well as those obtained from velocities or accelerations, although direct measurements are unlikely to be affected by transients or the false datum unless the data have been high-pass filtered. The technique is illustrated with reference to a simple analytical model for track deflection, which will now be introduced.

## A model for track deflection

**Figure 2.**
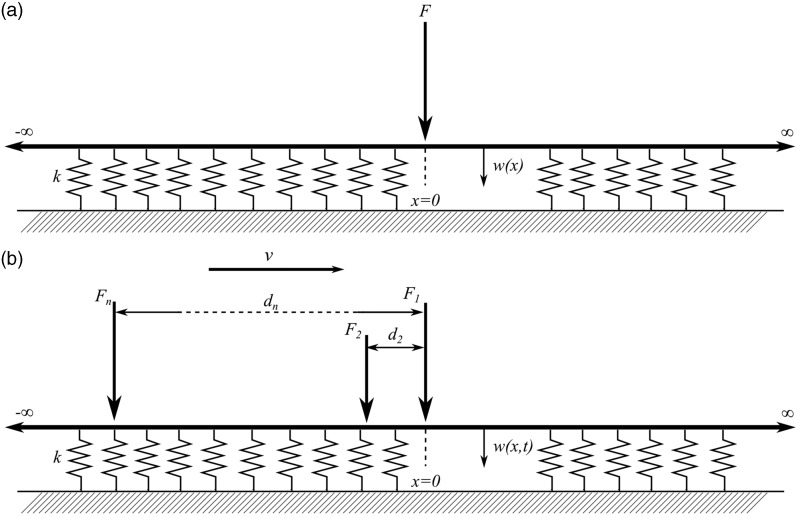
Beam on an elastic foundation subject to (a) a static point load and (b) a train of moving loads.

A simple and widely accepted model for the displacement of a railway track at low frequency is a beam on an elastic foundation.^19^ Neglecting inertial effects and track roughness, this can be used to model the deflection due to a single static load (Figure 2(a)), a moving load or a train of moving loads (Figure 2(b)).

The static displacement *w* at distance *x* along a beam with bending stiffness *EI* on an elastic foundation with a system support modulus (stiffness per unit length) *k* has the governing equation

(1) $$\begin{array}{c} \mathrm{EI}\frac{d^{4}w(x)}{{\mathrm{dx}}^{4}}+\mathrm{kw}(x)=0 \end{array}$$

The deflection due to a single load *F* acting at *x = *0 (Figure 2(a)) has the solution

(2) $$\begin{array}{c} w(x)=\frac{F}{2\mathrm{kL}}{\text{e}}^{-\frac{|x|}{L}}(\text{cos}(\frac{|x|}{L})+\text{sin}(\frac{|x|}{L})) \end{array}$$

where *L* is the characteristic length

(3) $$\begin{array}{c} L=\sqrt[{4}]{\frac{4\mathrm{EI}}{k}} \end{array}$$

A point on the track subjected to a load moving at a constant speed *v* experiences this deflection as a function of time *t = x*/*v*. The solution for the time-varying displacement of the track at a point due to a train of moving loads (Figure 2(b)), each separated from the first load by a distance *x_n_*, can be found by summing the effect of each wheel load

(4) $$\begin{array}{c} \begin{array}{c} w(t)=\sum_{n}\frac{F}{2\mathrm{kL}}{\text{e}}^{-\frac{v|t-t_{n}|}{L}}(\text{cos}(\frac{v|t-\frac{x_{n}}{v}|}{L})+\text{sin}(\frac{v|t-\frac{x_{n}}{v}|}{L})) \end{array} \end{array}$$

If measurements are made on the sleeper instead of on the rail, the sleeper deflection *w_s_* has the same pattern but with a reduced amplitude that depends on the ratio of the rail pad modulus *k_p_* to the total system support modulus *k*

(5) $$\begin{array}{c} w_{s}=w(1-\frac{k}{k_{p}}) \end{array}$$

## The distribution of track deflection

The stationary region in a displacement time history obtained from lineside measurements is expected to have a distribution similar to the track deflection obtained from the beam on an elastic foundation model. This means that at a randomly selected time or location, the probability *P* of the measured track displacement *w_m_* being at or below the at-rest position *w_r_* will be similar to the probability of the modelled displacements *w* being at or below zero for equivalent time periods or length of track.^a^

(6) $$\begin{array}{c} P(w_{m}\leq w_{r})=P(w\leq 0) \end{array}$$

*P* is the proportion of the time that the track spends at or below a given vertical position during the passage of a train. This means that the cumulative distribution function for the beam on an elastic foundation model can be used to determine values of probability suitable for identifying the at-rest position and classifying the range of track movement from displacement signals obtained from lineside measurements.

The cumulative distribution function for a variable distributed according to a function can be found analytically from the normalised inverse of that function^20^ or numerically using the product limit estimator.^21^ For simplicity, this analytical example uses the static solution for a beam on an elastic foundation subjected to a single point load, equation (2), rather than a train of loads, equation (4).

**Figure 3.**
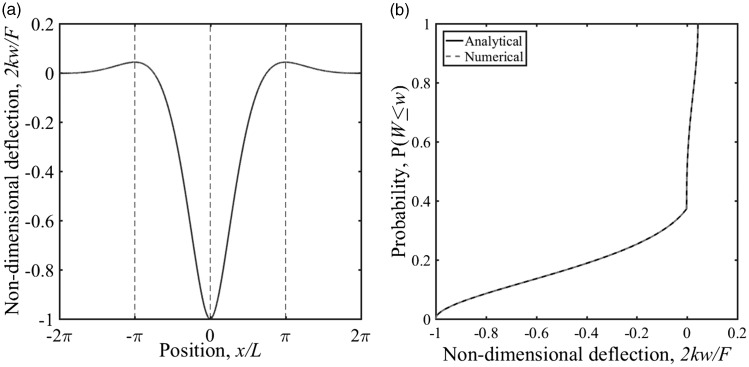
(a) Non-dimensional displacement *2 kw*/*F* at positions *x*/*L* along a beam on elastic foundation; the monotonic regions are separated using the dashed lines (b) distribution function for the region [0, 2π].

The inverse function *x*(*w*) for a function *w*(*x*) to be invertible it must be unique for each *x*. The beam on elastic foundation does not satisfy this condition (see Figure 3(a)); thus, the domain must be restricted to monotonic regions [*x*_1_*, x*_2_]. The solution for the beam on elastic foundation can be evaluated analytically over each region using integration by parts

(7) $$\begin{array}{c} \int_{w_{1}}^{w_{2}}x(w)\text{d}w=\int_{x_{1}}^{x_{2}}w(x)\frac{\mathrm{dw}}{\mathrm{dx}}\text{d}x\\ =[\mathrm{xw}(x){]}_{x_{1}}^{x_{2}}-\int_{x_{1}}^{x_{2}}w(x)\text{d}x \end{array}$$

and

(8) $$\begin{array}{c} \int_{x_{1}}^{x_{2}}w(x)\text{d}x=[\frac{F}{2k}{\text{e}}^{-\frac{|x|}{L}}\text{cos}(\frac{|x|}{L}){]}_{x_{1}}^{x_{2}} \end{array}$$

The cumulative distribution function for the entire function may be evaluated by integrating over all the regions.

For a single load, the boundaries of the monotonic regions occur at *|x|/L = nπ*, as shown for a unit maximum displacement of the beam in Figure 3(a). The cumulative distribution function for the region [0, 2π] has been evaluated analytically in Figure 3(b). Although it is possible to determine the distribution function for an entire train, it is far more onerous, as the boundaries for the monotonic regions will depend on the train geometry and the track system support modulus. The alternative is to discretise the solution and find the cumulative distribution function numerically using the Kaplan–Meier estimator.^21^ This has been done for the same region as the analytical solution in Figure 3(b). The result for the region [−2π, 0] and hence for [−2π, 2π] would be the same due to symmetry. Outside this region there is approximately zero deflection. The analytical and discrete cumulative distribution functions are the same.

The distribution has a distinct shape, a shallow section and then a change of gradient into a steep region associated with approximately zero deflection. This facilitates the use of the cumulative distribution function to estimate the at-rest position. In the remainder of this paper, the numerical rather than the analytical approach will be used. It allows different train geometries and the influence of the track system support modulus to be considered without the need to find the boundaries to the monotonic regions. The numerical process is required for obtaining the cumulative distribution function from lineside measurements.

## Theoretical at-rest position

The track deflection at a given time and location due to multiple wheel loads is a function of the vehicle geometry and the track system support modulus (equation (4)). The vehicle geometry determines where the loads are positioned relative to each other and the track modulus controls the size of the deflection bowl beneath each load,^11^ which also affects the interaction between adjacent loads. As both the vehicle geometry and the track stiffness affect the pattern of deflection, they will affect how those deflections are distributed. The numerical approach will now be used to explore the role of vehicle geometry and track stiffness in determining the distribution of track deflections and their influence on the probability value corresponding to the at-rest position *P*(*w* = 0). The results will be used to assess which probability value should be used for interpreting the at-rest position in lineside measurements.

**Figure 4.**
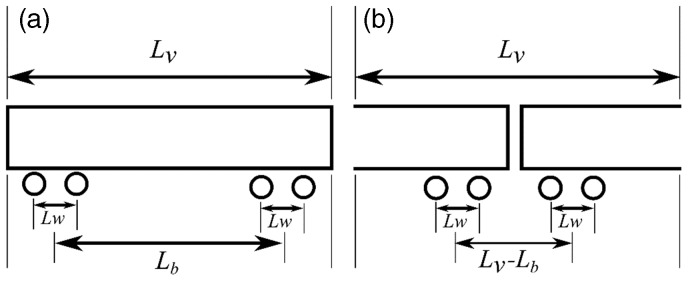
Simplified vehicle geometry defined for (a) a single vehicle and (b) two half vehicles.

Many trains comprise near-identical repeating vehicles or a have a primary repeating vehicle type with a common axle geometry, as illustrated in Figure 4(a). This will create periodic loading patterns within a train.^12,22^ The displacements within one period will be distributed in the same way as in others. It is convenient to define a period as one vehicle length containing two half vehicles as shown in Figure 4(b).

**Figure 5.**
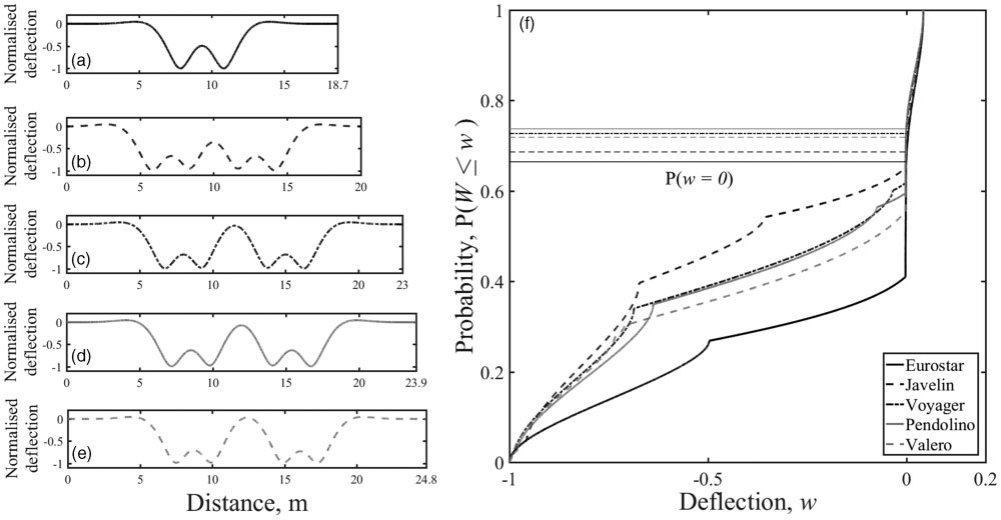
(a–e) Unit deflections for different vehicle types and (f) their associated cumulative distribution functions.

Table 1 summarises the primary vehicle geometries for a number of trains operating in the United Kingdom. Figure 5(a) to (e) shows the track displacement, normalised to a unit deflection, for each of these vehicle geometries, for a single period as defined in Figure 4(b), on a track with a characteristic length of *L* = 1 m (equivalent to *k* = 25 MN/m^2^ for track with a UIC 60 rail). The cumulative distribution functions found for each vehicle type are plotted in Figure 5(f).

**Table 1.** Primary vehicle geometries.

Train	Vehicle length, *L_v_*	Bogie spacing, *L_b_* (*L_v_* − *L_b_*)	Wheel spacing, *L_w_*
Eurostar	18.7	–	3.0
Javelin	20.0	14.2 (6.8)	2.6
Voyager	23.0	16.0 (7.0)	2.6
Pendolino	23.9	17.0 (6.9)	2.7
Valero	24.8	17.4 (7.4)	2.5

Figure 5 shows that there are differences in the cumulative distribution function for the different vehicle types when on a track with the same properties. The cumulative distribution function shows the proportion of time (or space) that the track is at or below a certain position. The probability value corresponding to the at-rest position is indicated. Vehicles that are shorter or have more wheels tend to cause the track to be below the at-rest position for a greater proportion of the train passage. The kinks in the curves correspond to the positions between adjacent wheels where the deflection does not fully recover to the at-rest position between wheels. These curves have a similar shape to the analytical result for a single load from Figure 3(b). The cumulative distribution functions gently slope, followed by a change in gradient to near vertical in the vicinity of the at-rest position.

**Figure 6.**
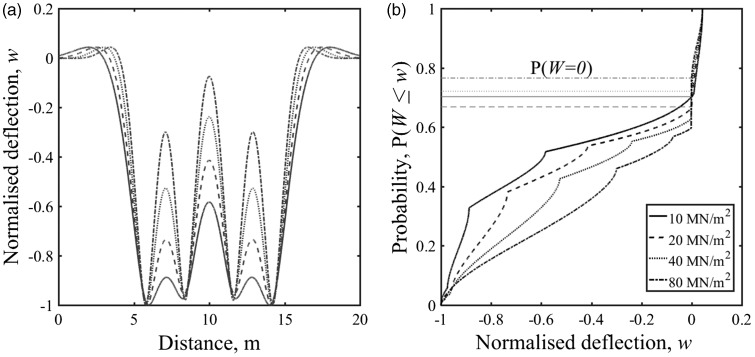
Effect of varying the track system support modulus on (a) track deflection and (b) the cumulative distribution function for track deflection for a Javelin.

The track system support modulus also affects the shape of the cumulative distribution function. Figure 6 shows the track displacement, normalised to give a unit maximum deflection, and the associated cumulative distribution functions for the Javelin vehicle geometry for various plausible track system support moduli. As the track modulus reduces, the deflection bowl associated with each wheel widens. This causes more interaction or less recovery between wheels on track with a lower support stiffness, resulting in the track being at or below the at-rest position for different proportions of the train passage.

**Figure 7.**
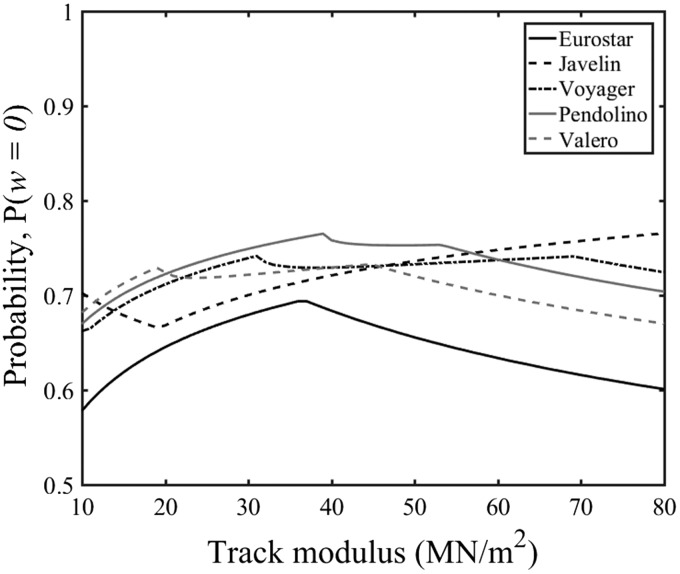
Probability corresponding to the at-rest position of the track for different vehicle geometries and a plausible range of track moduli using the beam on an elastic foundation model.

In Figures 3(a), 5(f) and 6(b) the region of the cumulative distribution function associated with the at-rest position *w* = 0 is very steep compared with the other regions. This means that the region associated with the at-rest position is clearly identifiable in the cumulative distribution function. Each cumulative distribution function has a different exact probability associated with the at-rest position, *P*(*w* = 0). Figure 7 shows how the exact probability corresponding to the at-rest position varies with track support stiffness for different train types. However, for the trains and range of stiffness considered, the probability associated with the at-rest position varies by less than 0.1 and generally lies between 0.6 and 0.8. A single value within that range may therefore be used to determine the at-rest position from measurements. For example, from Figure 7 the minimum, mean and maximum possible values for a Javelin with plausible values of track system support modulus between 10 and 60 MN/m^2^ are *P* = 0.66, 0.70 and 0.75. All of these values lie within the steep region of the cumulative distribution function and have been used later for analysis.

The length of the steep region (Figure 5(f)) suggests that there are a range of values that approximately correspond to the at-rest position. This means that any value in the steep region could be selected for a particular train type and range of support conditions to estimate the at-rest position from measurements. Those estimates will not be sensitive to imprecision or variation in the value of probability used. This is beneficial for the reliability and implementation of the method as it can be challenging to obtain a precise measurement of the track system support modulus, particularly in advance of characterising the range of deflection. Furthermore, provided the overall pattern of track deflection conforms to the approximate shape given by the beam on an elastic foundation model, it is expected that the distribution could still be used to interpret data from poorly supported or voided sleepers.

**Figure 8.**
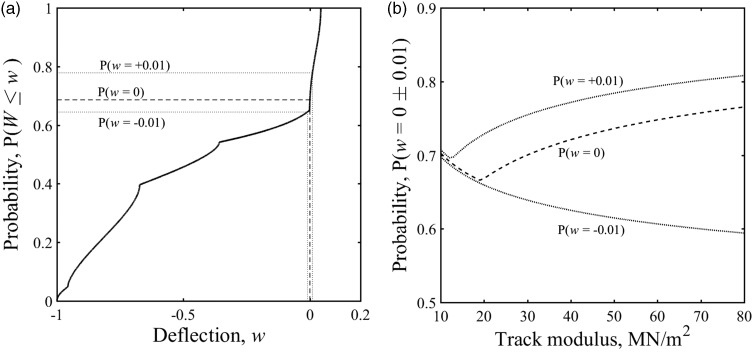
(a) Change in probability value associated with an over- or under-estimate of the at-rest position by 1% of the downward deflection for a Javelin trainset on track with *L* = 1 m. (b) Probability values for P(*w = *0 ± 0.01) for a Javelin on a track with a range of plausible track moduli.

The sensitivity of the approach to variations in the value of probability used to identify the at-rest position is shown in Figure 8 for a Javelin vehicle. Figure 8(a) shows the probability associated with an over- or under-estimate of the at-rest position by 1% of the downward deflection on a track with a characteristic length of *L* = 1 m as before. There is quite a significant change in probability for a small change in the at-rest position, owing to the steepness of the function. The analysis has been extended for a range of plausible track moduli in Figure 8(b). The thick dashed line shows the probability values for P (*w = 0*) and the thin dotted lines on either side show the values for P (*w* = ± 0.01), corresponding to a small error in the estimate of the at-rest position.

**Figure 9.**
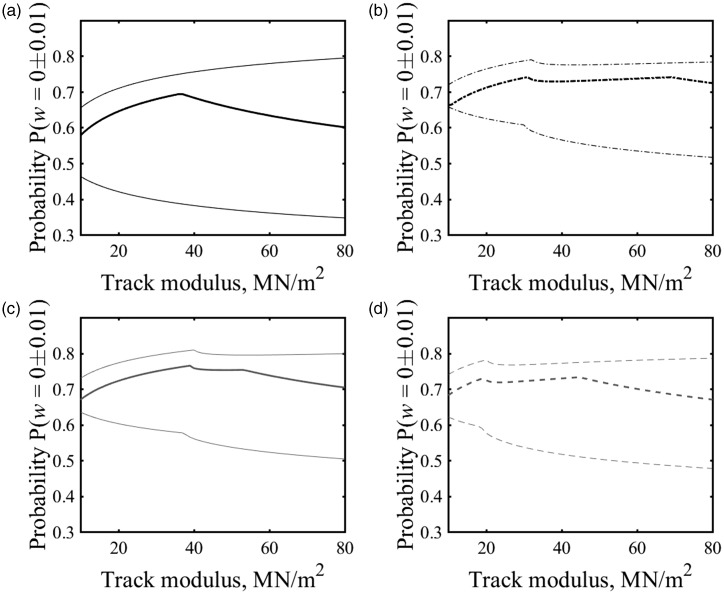
Probability values for P (*w = *0 ± 0.01) for (a) Eurostar, (b) Voyager, (c) Pendolino and (d) Valero on a track with a range of plausible track moduli.

Probabilities of the track being near the at-rest position for the other train types studied are shown in Figure 9. The significance of the change in probability depends on both the vehicle geometry and the track system support modulus. For the vehicles studied, on track with a track system support modulus *k* > 20 MN/m^2^, a change in the probability assumed to correspond to the at-rest position of Δ*P* ≈ ± 0.1 is unlikely to change the estimate of the at-rest position significantly. This range Δ*P* ≈ ± 0.1 covers the range of probability values corresponding to the at-rest position from Figure 8. The effect of overestimating the probability is not particularly sensitive to variations in the track modulus, while the effect of underestimating the probability increases with track stiffness.

## Measured at-rest position

The analysis of the previous sections has shown that the cumulative distribution function has a distinct shape, with an abrupt change in gradient to a steep region associated with the at-rest position of the track. This can be used to estimate the at-rest position in measured track displacement data, and subsequently to correct the shift in that datum in data obtained from velocity or acceleration measurements. This estimate is not sensitive to the probability value used to identify the at-rest position as long as it is in the steep region. Provided data from measurements have been filtered appropriately, a stationary region can usually be identified within a track displacement signal. This region can be truncated into an integer number of vehicle periods thereby avoiding the transients seen in Figure 1. The cumulative distribution function can be evaluated numerically for the truncated region and the at-rest position can be estimated. Evaluating measurements for several vehicles will implicitly average the data.

**Figure 10.**
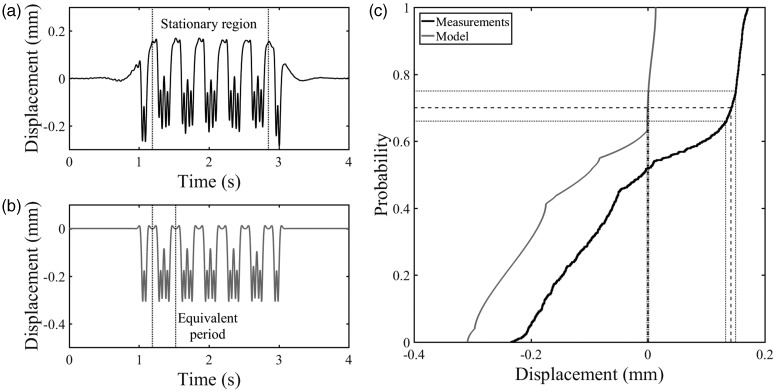
Displacement data for a well-performing sleeper (a) obtained from measured geophone data, sampled at 500 Hz, filtered with high- and low-pass filters with 2 and 40 Hz cut-offs, (b) obtained using the beam on an elastic foundation model and (c) the cumulative distribution functions for the measured and modelled data.

The utility of the method will now be assessed using deflection data obtained for a sleeper on a well-performing track and a sleeper above a void, in both cases for a Javelin train. Figure 10(a) shows the sleeper displacement data obtained using a geophone for a six-vehicle train moving at 60.2 m/s. Data were sampled at 500 Hz and filtered using high- and low-pass filters with cut-off frequencies of 2 and 40 Hz respectively, covering the range of frequencies significant for track deflection. The track had a system support modulus of 36.3 MN/m^2^, determined using the method described in Le Pen et al.^11^ The sleeper can be seen to move by about 0.35 mm. Figure 10(b) shows the results for the same train modelled using the beam on an elastic foundation approach. The cumulative distribution functions for the stationary region indicated in the measurements (Figure 10(a)), and for the equivalent period from the model are shown in Figure 10(c). Generally, the cumulative distribution function of the measurements is similar to that obtained from the model. There is a steep region in the measurements likely to be associated with the at-rest position, which can be used to estimate the at-rest position. The location of the at-rest position in the measurements has been estimated using probability values of *P* = 0.66, 0.70 and 0.75, from before, giving 0.135, 0.145 and 0.150 mm, respectively.

**Figure 11.**
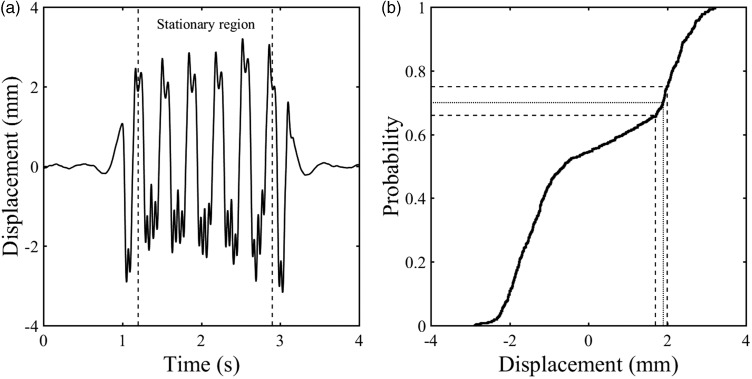
(a) Displacement data of a voided sleeper obtained using a MEMS accelerometer, sampled at 333 Hz and filtered with high- and low-pass filters with 2 and 40 Hz cut-offs and (b) cumulative distribution function for that displacement data.

Figure 11(a) shows the deflection data obtained using a MEMS accelerometer from a different site to Figure 10, for a sleeper that moved about 4 mm under passing trains, suggesting the presence of a void beneath it. Data were sampled at 333 Hz and filtered with high- and low-pass filters with cut-off frequencies of 2 and 40 Hz, respectively. The sample rate was reduced to allow a greater number of transducers to be used with a single data acquisition system. The cumulative distribution function for the stationary region in Figure 11(a) is shown in Figure 11(b). A steep part is likely to be associated with the at-rest position and can be used to estimate it. The same probability values used previously gave estimates of 1.7, 1.9 and 2.0 mm for the at-rest position. The shape of the cumulative distribution below the at-rest position is also of significance. There are two distinct regions with constant gradients. Between *P* ≈ 0.03 and 0.50 the cumulative distribution function is quite steep. The gradient reduces between 0.5 and 0.65. This shape differs from the result that would be obtained from the model even with very soft track support, *k* ≤ 10 MN/m^2^ (see Figure 6(b)).

Practically it would be useful to be able to select a single probability value to represent the at-rest position as this would enable rapid analysis of deflection data. In the results shown here, the differences between the at-rest positions obtained from the measurements using different probability values were greater than expected from the theoretical analysis. However, the differences between the estimates of the at-rest position found using *P* = 0.66 and 0.70 were larger than between *P* = 0.7 and 0.75. This suggests that it may be more reliable to select a value that marginally overestimates the probability associated with the at-rest position. This means it is more likely to fall within the steep region in the cumulative distribution function for track deflection where imprecision over the value chosen has a small effect on the estimate of the at-rest position obtained.

## Characterising deflection

The cumulative distribution function provides a basis for characterising the typical range of deflection, which, with an estimate of the at-rest position, can be categorised as either downward deflection or uplift. Differentiating between upward and downward movement from the at-rest position is important. Most of the track deflection is expected to be in the downward direction, and deflection beneath a wheel load is commonly used to assess the track stiffness. Uplift effects are expected to be small, around 4% of the downward deflection according to the beam on an elastic foundation model (equation (2)); a greater uplift may indicate non-typical behaviour. This can be investigated by selecting probability values that represent typical upper and lower limits of track movement. Although the pattern of track deflection tends to repeat for similar vehicles, there will be variation, owing to differences in vehicle weight, occupancy and the dynamic behaviour of each wheel, including the response to wheel flats. This means that it would be inappropriate to select either the maximum upward or downward deflection (i.e. at *P* = 1 or 0) to characterise the typical range of movement. Values that encompass the majority of the typical range of movement yet avoid irregular movements from wheel impacts are more suitable.

**Figure 12.**
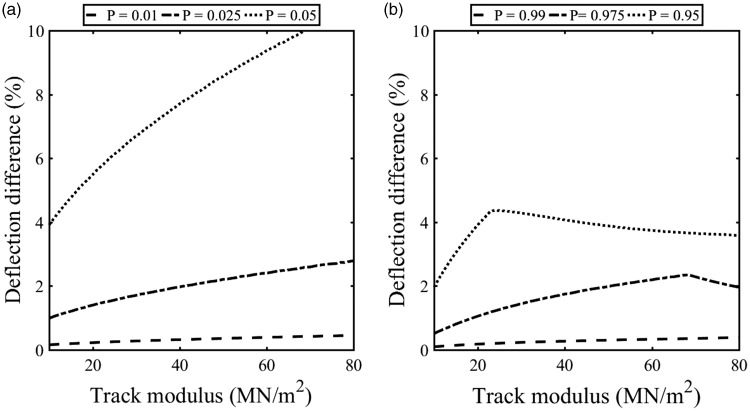
Difference between the exact and approximate deflection determined using confidence intervals (a) for the downward deflection and (b) for the uplift.

The cumulative distribution function for the beam on an elastic foundation model can provide guidance for selecting appropriate values representative of typical minimum and maximum deflections. Figure 12(a) shows the difference between the exact (*P* = 0) and approximate downward deflection found using probabilities of *P* = 0.01, 0.025 and 0.05 expressed as a percentage of the exact downward deflection. Figure 12(b) shows corresponding results for the difference between the exact (*P* = 1) and approximate uplift determined using *P* = 0.99, 0.975 and 0.95, expressed as a percentage of the exact uplift. For clarity, these results are shown for the Javelin vehicle type for a range of plausible track system support moduli. These show which probabilities may give a reasonable approximation to the exact value of downward deflection or uplift. The difference between the theoretical deflection and the corresponding approximation is less than 2% for *P* = 0.025 and 0.975; similar results were obtained for the other train types studied. In theory, the amount of uplift should be small; hence, the associated absolute error will be small unless there are significant uplift effects.

**Figure 13.**
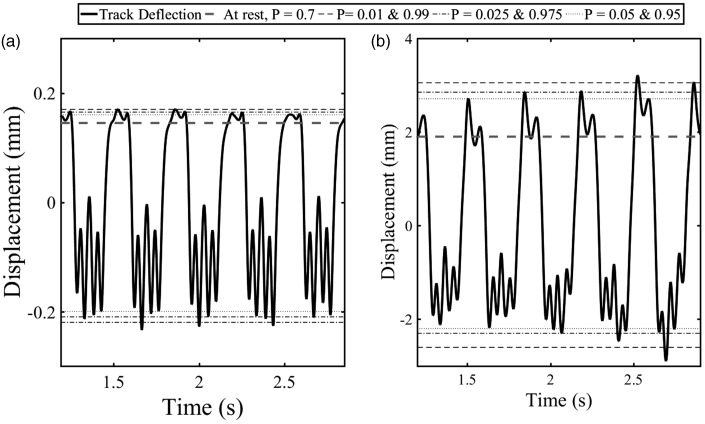
Identification of the typical characteristic upward and downward deflection and at-rest position for (a) track with well-performing sleeper and (b) track with voided sleeper.

Figure 13(a) and (b) shows the results of applying the probability values used in Figure 12 to the stationary region of the displacement data from Figure 10(a) and Figure 11(a), respectively. The positions associated with the at-rest level and the typical extents of movement have been identified. These can be used to find the total range of movement, the characteristic downward deflection and the amount of uplift. The at-rest position found using *P* = 0.7 in Figure 13 is close to where it would have been identified by inspection. The downward deflection was identified using *P* =0.01, 0.025 and 0.05 and the uplift using *P* = 0.99, 0.975 and 0.95. Adopting such values rather than the maximum and minimum should help to mitigate the effects of irregular or extreme movements that could be present for a small part of the train passage.

The results obtained as shown in Figure 13 are given in Table 2. Those found using *P* = 0.01 and 0.99 are the most influenced by the extremes of movement. Both *P* = 0.025 and 0.975 and *P* = 0.05 and 0.95 give reasonable estimates for the typical total range of movement. Figure 13 indicates that *P* = 0.025 and 0.975 would be more representative of the total range of movement in the absence of variable or irregular track movements. Analysis was carried on the entire stationary region of the displacement signal, to provide a result representative of the entire train.

**Table 2.** Typical characteristic downward and upward track deflection (in mm) identified using different probability values from displacement data of a track with well-performing sleeper and a track with voided sleeper from Figure 13.

Probability values	Well-performing sleeper			Voided sleeper		
	Downward (mm)	Upward (mm)	Total (mm)	Downward (mm)	Upward (mm)	Total (mm)
0.010, 0.990	0.36	0.03	0.39	4.50	1.15	5.65
0.025, 0.975	0.35	0.02	0.37	4.20	0.95	5.15
0.050, 0.950	0.34	0.02	0.36	4.10	0.81	4.91

**Figure 14.**
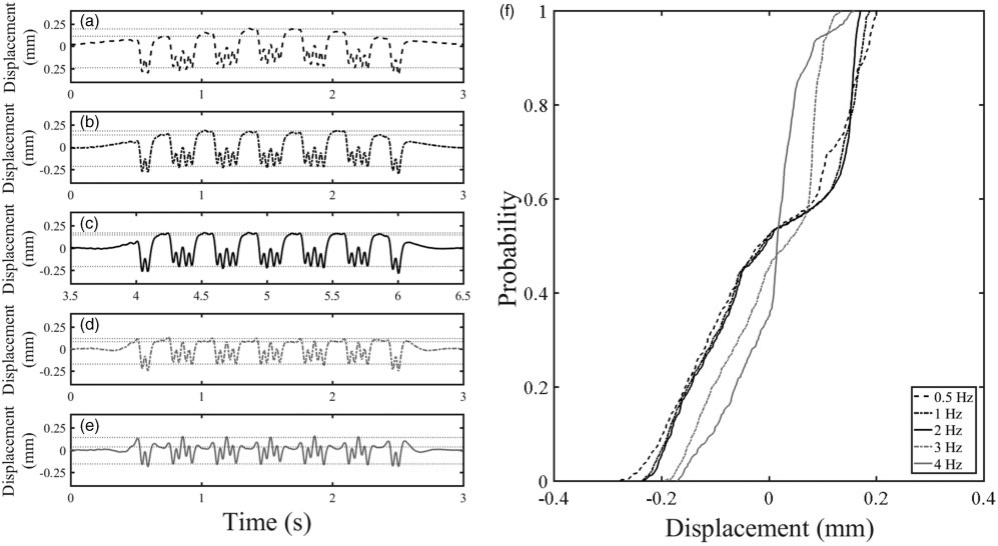
Sensitivity of the displacement obtained from velocity measurements to the cut-off frequency used for the high-pass filter. This is set at (a) 0.5, (b) 1, (c) 2, (d) 3 and (e) 4 Hz, where the vehicle passing frequency is around 3 Hz. (f) The effect on the cumulative distribution function of these displacements.

This analysis is based on the assumption that displacements from velocity or acceleration data were filtered appropriately to obtain a stationary region within the signal. This is influenced by the cut-off frequency used for the high-pass filter. If the cut-off frequency is too low the signal will be affected by low-frequency drift; if it is too high, some of the frequency content relevant to track deflection will be filtered out. The influence of the filter cut-off is shown in Figure 14. Here displacements have been obtained from the velocity data used in Figure 10 with cut-off frequencies of 0.5, 1, 2, 3 and 4 Hz for the high-pass filter (Figure 14(a) to (e), respectively). A cut-off frequency of 40 Hz was used for the low-pass filter in all cases. The displacements corresponding to P = 0.025, 0.7 and 0.975 have been marked and estimates of the downward, upward and total deflection are recorded in Table 3. Results were also obtained using cut-off frequencies of 2.25 and 1.5 Hz. These were similar to those obtained using a 2 Hz cut-off (Table 3), suggesting that for this train, 1/2, 2/3 and 3/4 of the vehicle passing frequency (3 Hz) are suitable as a high-pass cut-off.

**Table 3.** Typical characteristic downward and upward track deflections identified using P = 0.025, 0.7 and 0.975 for different high-pass filter cut-off frequencies.

High pass filter cut-off frequency (Hz)	Sleeper movement		
	Downward (mm)	Upward (mm)	Total (mm)
0.5	0.35	0.08	0.43
1	0.35	0.05	0.40
1.5	0.35	0.03	0.38
2	0.35	0.02	0.37
2.25	0.35	0.02	0.37
3	0.25	0.04	0.29
4	0.19	0.11	0.30

In Figure 14(a) and (b), for cut-off frequencies of 0.5 and 1 Hz, the result is affected by low-frequency drift. No single level can be associated with the at-rest position and the total deflection is greater than when a 2 Hz cut-off was used (although in this case, the downward deflections were the same). In Figure 14(d) and (e), where the cut-off frequencies were greater than or equal to the vehicle passing frequency of 3 Hz, the relevant frequency content is beginning to be filtered out resulting in a reduced apparent deflection. Changing the filter cut-off frequency, such that the drift begins to affect the signal or that information is filtered out, changes the cumulative distribution function (Figure 14(f)). This highlights the importance of appropriate filtering for obtaining a stationary region for analysis.

## Applications

**Figure 15.**
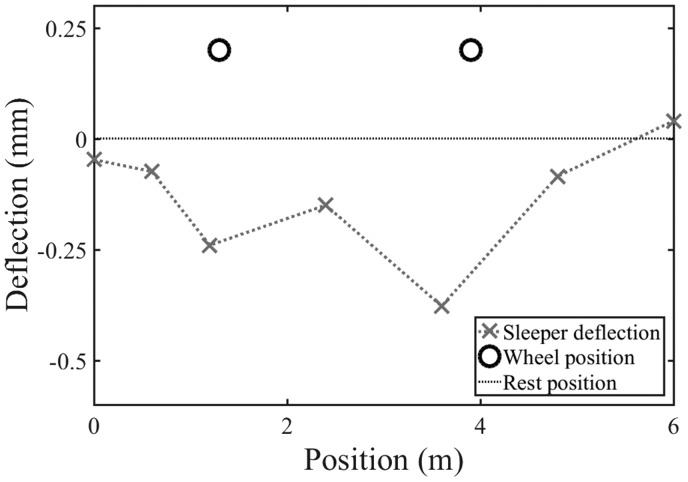
Visualisation of the instantaneous deflection bowl beneath a bogie of a Javelin train using data from geophone measurements.

Lineside measurements are routinely made using an array of sensors covering a length of track. Normally, such sensors will be placed at the same ‘level’ along the track, e.g. all on the sleepers or all on the rail, and their signals would be recorded using a shared data acquisition system so the data have a common timestamp. The ability to estimate and correct the shift in the at-rest position allows data from multiple transducers to be aligned to a common vertical datum relative to the rail. This enables visualisation of the track movement and deformed shape as a train passes along the track. This approach has been used to obtain a visualisation of the instantaneous deflection bowl beneath the wheels of a Javelin bogie in Figure 15.

**Figure 16.**
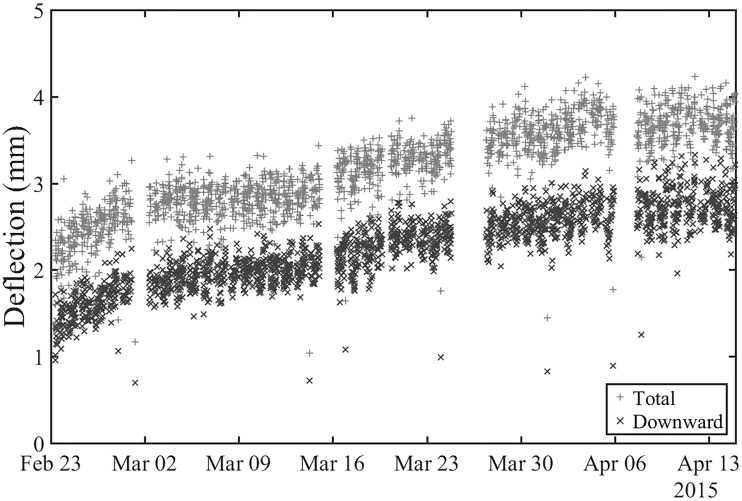
Deflection of a single sleeper due to passing trains showing deterioration in performance owing to proximity to the voided sleeper.

The total range of movement, characteristic downward deflection and uplift may also be used as a measure of how the track is performing during the passage of a train for condition monitoring purposes. Displacement data from lineside measurements can be interpreted and characterised automatically at regular intervals over a period of time using the method proposed in this paper, to provide a record of how the performance of the track changes with trafficking. Figure 16 shows the total deflection (between *P* = 0.975 and 0.025) and characteristic downward deflection (between *P* = 0.70 and 0.025) obtained from acceleration measurements made on a sleeper over a period of seven weeks. The performance was deteriorating at a high rate owing to a nearby voided sleeper. This illustrates how the analysis method can be used to provide data suitable for condition monitoring. A total of 600 train passages were analysed to produce the results as shown in Figure 16, which would not be feasible without the automated process described here.

## Conclusions

This paper has shown how the cumulative distribution functions can be used to identify the at-rest position in track deflection data obtained from velocity or acceleration measurements, overcoming artefacts from signal processing. The cumulative distribution function has a distinct shape, a steep region being associated with the at-rest position. This means that the at-rest position can be identified statistically using a probability value that is equivalent to the proportion of time the track spends at or below a given position during the passage of a train.

The shape of the cumulative distribution function means the estimate of the at-rest position is not sensitive to the exact value selected for this probability. This means that a single probability value, e.g. *P* = 0.7, may be used to find the at-rest position in data for a range of train types and track properties, facilitating automated analysis for the condition monitoring purposes, provided that value lies within the steep region in the cumulative distribution function. Other probability values, e.g. *P* = 0.975 and 0.025, can be used to determine the typical upward and downward extents of movement, thus characterising the range of total, upward and downward track deflection. This approach accounts for variations in load between wheels and reduces the influence of irregular track movements on the result, giving a result representative of the train passage.

This type of analysis can be implemented on a computer, enabling track deflection signals to be interpreted automatically. An example using site monitoring data was presented to illustrate how processed data from multiple transducers can be aligned to a common datum, providing metrics for performance monitoring. Changes in deflection over time can easily be identified, in a way that would not be feasible without such an analysis technique. Thus, the proposed approach has major potential for use in automatic condition monitoring of railway tracks.

## Note

a Note that, although discussed in terms of probability, this should not be taken to imply there is any stochastic element to the model used, which remains mechanistic.
